# Correction: Impact of containment measures on community mobility, daily confirmed cases, and mortality in the third wave of COVID-19 epidemic in Myanmar

**DOI:** 10.1186/s41182-022-00487-4

**Published:** 2022-12-21

**Authors:** Ye Minn Htun, Tun Tun Win, Nyan Htet Shan, Zin Thu Winn, Kaung Si Thu, Nyan Lin Maung, Pyae Phyo Aung, Htun Aung Kyaw, Hpone Pji Kyaw, Yan Naing Myint Soe, Myint Myat Ko, Zin Ko Aung, Kyaw Thiha Aung, Yan Paing Chit Lwin, Wai Yan, Phyo Tayza Soe, Kyaw Myo Tun

**Affiliations:** 1 Department of Prevention and Research Development of Hepatitis, AIDS and Other Viral Diseases, Health and Disease Control Unit, Nay Pyi Taw, 15011 Myanmar; 2 Department of Preventive and Social Medicine, Defence Services Medical Academy, Mingaladon, Yangon Myanmar; 3 Outpatient Department, No. 1 Military Hospital (500 Bedded), Meiktila, Mandalay Myanmar; 4 Department of Research and Development, Defence Services Medical School, Hmawbi, Yangon Myanmar

**Correction: Tropical Medicine and Health (2022) 50:23** **https://doi.org/10.1186/s41182-022-00413-8**

Following publication of the original article [1], it was brought to our attention that the Competing Interests section was incorrect. It should read as follows:

TTW, PPA, HAK, HPK, MMK, ZKA, KTA, YPCL, WY, PTS and KMT are public health specialists of the Department of Preventive and Social Medicine, Defence Services Medical Academy. NLM is a public health specialist of the Department of Research and Development, Defence Services Medical School. None of the other authors have any competing interests to declare.
